# Correlation Between Salivary Cortisol Levels and Diurnal Variation in Spirometric Parameters in Apparently Healthy Adults

**DOI:** 10.7759/cureus.71493

**Published:** 2024-10-14

**Authors:** Tanuj Mathur, Yoshita Rao Annepu, Perugu Damodara Krishna Chaitanya, Rajiv Ranjan, Dileep K Verma, Narsingh Verma, Sandeep Pandey, Ranjana Singh

**Affiliations:** 1 Department of Physiology, Jawaharlal Nehru Medical College, Aligarh Muslim University, Aligarh, IND; 2 Department of Physiology, King George's Medical University, Lucknow, IND; 3 Department of Medicine, Rangaraya Medical College, Kakinada, IND; 4 Department of Biochemistry, King George's Medical University, Lucknow, IND

**Keywords:** diurnal variation, fef25-75%, fev1, fvc, pefr, salivary cortisol, spirometry

## Abstract

Background: This study investigated diurnal variations in spirometric parameters and their correlation with salivary cortisol levels among apparently healthy adults.

Methods: Forty subjects, aged 18-22, were assessed. Spirometric parameters, including forced vital capacity (FVC), forced expiratory volume in one second (FEV1), forced expiratory flow during 25%-75% of expiration, and peak expiratory flow rate, were measured in the morning, afternoon, and evening. Salivary cortisol levels were also measured at these intervals. Statistical analyses included calculating means, standard deviations, confidence intervals, and Pearson correlation coefficients.

Results: The mean FEV1 values were 2.8325 L in the morning, 2.9263 L in the afternoon, and 2.9543 L in the evening, slightly increasing throughout the day but with overlapping confidence intervals, suggesting no statistical significance. The FEV1/FVC ratios displayed a similar pattern. FVC values decreased slightly from morning to evening, but these changes were not statistically significant. Pearson correlation analysis revealed a strong positive correlation between salivary cortisol and FVC (r = 0.829, p = 0.055), a moderate negative correlation with FEV1 (r = -0.649, p = 0.038), and a strong negative correlation with the FEV1/FVC ratio (r = -0.730, p = 0.040).

Conclusion: Spirometric parameters exhibited minor diurnal variations, with no statistically significant changes for most measures except for salivary cortisol levels, which significantly decreased throughout the day. Significant correlations were observed between salivary cortisol and certain spirometric parameters.

## Introduction

The assessment of respiratory function through spirometry is a cornerstone of pulmonary diagnostics, providing critical insights into the mechanical properties and overall health of the respiratory system [1]. Spirometric parameters, such as forced vital capacity (FVC) and forced expiratory volume in one second (FEV1), are essential for diagnosing and monitoring respiratory conditions, ranging from asthma and chronic obstructive pulmonary disease (COPD) to restrictive lung diseases. These parameters are influenced by various intrinsic and extrinsic factors, including age, sex, body composition, environmental conditions, and, notably, circadian rhythms [2].

Circadian rhythms are endogenous, entrainable oscillations that cycle approximately every 24 hours and regulate numerous physiological processes, including sleep-wake cycles, hormone secretion, and metabolic functions [3]. The respiratory system is not immune to these rhythms; previous studies have indicated that pulmonary function can exhibit diurnal variations, with potential implications for the timing and interpretation of spirometric assessments. Understanding these variations is crucial, as they can affect the accuracy and reliability of spirometric measurements, potentially leading to misinterpretations if the timing of the test is not considered [4,5].

Cortisol, a glucocorticoid hormone produced by the adrenal cortex, is a key player in the body's circadian system [6]. It follows a well-documented diurnal pattern, peaking in the early morning shortly after awakening and gradually declining throughout the day. This hormone is integral to a wide array of physiological processes, including the regulation of metabolism, immune response, and stress [7]. Cortisol also significantly affects the respiratory system; it can influence airway inflammation and bronchial responsiveness, which are critical factors in respiratory health and disease [8]. The interplay between cortisol levels and pulmonary function is complex and not fully understood, but evidence suggests that cortisol's anti-inflammatory properties may play a role in modulating respiratory parameters [9].

Despite the established diurnal pattern of cortisol and its potential impact on respiratory function, there is limited research exploring the direct correlation between cortisol levels and spirometric parameters throughout the day in healthy individuals. Most studies have focused on pathological conditions, leaving a gap in our understanding of how these variables interact in a healthy population [10-12]. Investigating this relationship is important for several reasons: it could provide insights into the optimal timing for spirometric testing, improve the accuracy of baseline measurements, and enhance our understanding of the physiological mechanisms underlying respiratory function. In this context, we aim to investigate the diurnal variation in spirometric parameters, specifically FVC, FEV1, and FEV1/FVC ratio, among apparently healthy adults and seek to examine the correlation between these spirometric parameters and salivary cortisol levels measured at different times of the day.

## Materials and methods

Study design

This was an observational, cross-sectional study evaluating circadian rhythm in spirometric parameters and salivary cortisol levels. Individuals aged 18-35 years of both genders were recruited from the university campus. Participants were age- and sex-matched. Spirometric assessments were conducted in the Pulmonary Function Test Lab, Department of Physiology, and salivary cortisol levels were measured using enzyme-linked immunosorbent assay (ELISA) in the Molecular Biology Lab, Department of Biochemistry, King George’s Medical University, Lucknow. The study was conducted from July 30, 2019, to July 30, 2020. Ethical clearance was granted by the University's Institutional Review Board, with the registration number ECR/262/Inst/UP/2013/RR-16 being assigned to this study.

Sampling method

The subjects underwent venipuncture sampling to get their blood samples, which were then put in ethylenediaminetetraacetic acid vials and stored at 4°C until further processing to avoid deterioration.

Sample size calculation

The formula below was used to determine the sample size for the current investigation [13]

 Z^2^_1 - α/2 _p(1 - p) / d^2^

where Z^2^_1-α/2_ is standard normal variate (1.96 at 5% type 1 error, p < 0.05; 2.58 at 1% type 1 error, p < 0.01). As in most studies, p values are considered significant at 0.05; hence, 1.96 is used in the formula. The expected proportion in the population is based on previous studies or pilot studies. d is the absolute error or precision, which is determined by the researcher. The report has calculated that a sample size of 40 is required for the study.

Inclusion criteria

The research subjects appeared to be young people (18-35 years) in good physical condition without a history of allergies, surgeries, respiratory conditions, or chest anomalies.

Exclusion criteria

The study excluded anyone who had experienced any unusual events within the past month, including pregnancy, use of tobacco or alcohol, acute respiratory infection, recent heart or pulmonary disease, leaking while under stress, states of dementia or confusion, and anyone who had not given their consent.

Anthropometric measurements

To reduce examiner bias, all assessments were performed by a single examiner. Using a nonelastic tape measure, height is determined to the closest centimeter. A digital portable scale calibrated daily is used to measure weight. The formula to calculate BMI is BMI = weight (kg) / height (m^2^). The waist circumference is measured midway between the iliac crest and the lowest rib. Hip width is the measurement point for hip circumference. Waist circumference divided by hip circumference yields the waist-hip ratio. Waist circumference divided by height yields the waist-to-height ratio. After five minutes of rest, blood pressure is measured with an Omron blood pressure monitor (model JPN1-HEM7200; Omron Healthcare Co., Ltd., Kyoto, Japan).

Pulmonary function test

Spirometry was performed using computerized spirometry. Instructions and demonstrations were provided. Tests were conducted in a standing position after a 10-minute rest, with a nasal clip used to block nostrils. Each test was repeated thrice with a five-minute rest between tests, and the best reading was recorded. Spirometric parameters included forced expiratory flow during 25%-75% of expiration (FEF25-75%), forced expiratory flow at 50% (FEF50), FEV1, FEV1/FVC, FVC, and peak expiratory flow (PEF). Measurements were taken at 8 AM, 12 PM, and 4 PM.

Saliva collection and analysis

Saliva samples were collected in coded vials at 8 AM, 12 PM, and 4 PM. Participants washed their hands and rinsed their mouths before collection. Samples were centrifuged at 3,000 rpm for 15 minutes and analyzed using ELISA kits from Applabs Bio Sciences, Lucknow, India (DiaMetra catalog no. DK0001).

ELISA study

The ELISA method involved cortisol competition with horseradish peroxidase-conjugated cortisol for antibody binding, followed by a colorimetric reaction to quantify cortisol concentration using an ELISA Kit (Human cortisol ELISA Kit, Elabscience, Houston, TX). For the cortisol ELISA study, saliva samples from 40 healthy individuals registered in the study were taken. Calibrators with cortisol concentrations of 0, 10, 50, 150, and 500 ng/mL were used. Spectrophotometer readings of the samples were measured at 450 nm against a reference wavelength of 620-630 nm within five minutes taken in Varioskan LUX Multimode Microplate Reader (Thermo Fisher, Waltham, MA).

Statistical analysis

Data were analyzed using SPSS version 20.0 (IBM Corp., Armonk, NY). Results were presented as means ± standard deviation, percentages, and tables. The Kolmogorov-Smirnov Z test checked data normality. Repeated measures analysis of variance compared continuous variables across different periods. Pearson's correlation test evaluated the relationship between salivary cortisol and spirometric parameters. A p value of ≤0.05 was considered statistically significant.

## Results

**Figure 1:**
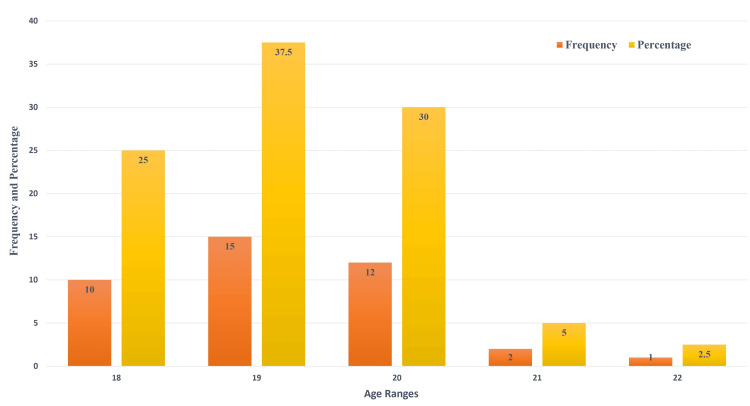
Age distribution of the study population

**Figure 2:**
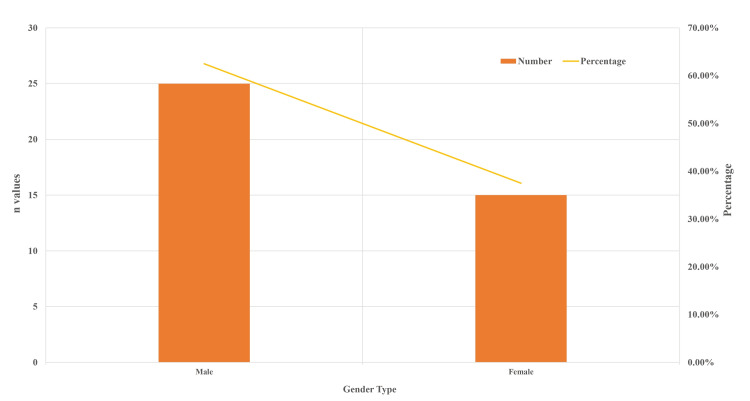
Gender distribution of the study population

**Figure 3:**
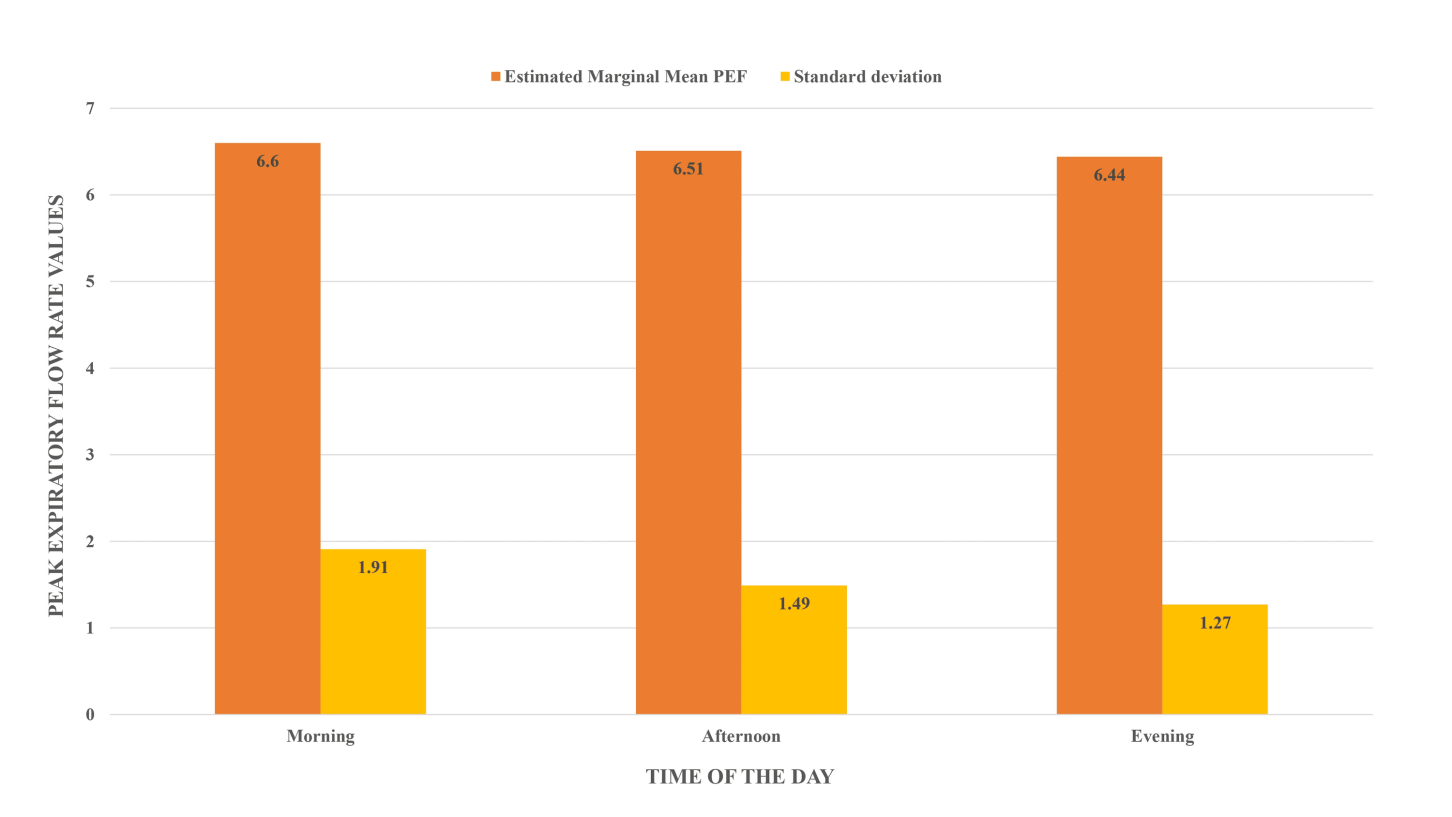
PEFR values at different time intervals PEF: peak expiratory flow; PEFR: peak expiratory flow rate

Figure 1 depicts the age distribution of study subjects in years. A total of 40 subjects were assessed, with a maximum of 22 years and a minimum of 18 years. The mean age of the study subjects was 19.22 + 97 years. When considering the age group of study participants, the majority of them were 19 and 18-22 years old. Figure 2 shows the genderwise distribution of study participants, where 62.5% were males and 37.5% were females. Most of the study participants were males (62.5%), while the remaining 37.5% were females. Figure 3 shows mean peak expiratory flow rate (PEFR) values assessed in the morning (6.60 + 1.91), afternoon (6.51 + 1.49), and in the evening (6.44 + 1.27) in the study population and the maximum and minimum values of PEFR at different time intervals. The highest value was noted in the morning, followed by the afternoon and evening.

The mean FEV1 values were 2.8325 L in the morning, 2.9263 L in the afternoon, and 2.9543 L in the evening, with standard deviations of 0.62372, 0.55670, and 0.52285, respectively (Table 1). The 95% confidence intervals for FEV1 were 2.633-3.032 in the morning, 2.748-3.104 in the afternoon, and 2.787-3.121 in the evening, slightly increasing throughout the day. The mean FEV1/FVC ratios were 0.8378 in the morning, 0.8605 in the afternoon, and 0.8675 in the evening, with standard deviations of 0.06881, 0.06350, and 0.05109, respectively. The 95% confidence intervals for the FEV1/FVC ratio were 0.816-0.860 in the morning, 0.840-0.881 in the afternoon, and 0.851-0.884 in the evening, indicating a trend of increasing ratios throughout the day.

**Table 1:** Evaluation of FEV1 and FEV1/FVC ratio at different time intervals FEV1: forced expiratory volume in one second; FVC: forced vital capacity

Time of day	Measure	Mean	Standard deviation	N	Standard error	95% confidence interval (lower bound to upper bound)
Morning	FEV1 (L)	2.8325	0.62372	40	0.099	2.633-3.032
Morning	FEV1/FVC (%)	0.8378	0.06881		0.011	0.816-0.860
Afternoon	FEV1 (L)	2.9263	0.55670		0.088	2.748-3.104
Afternoon	FEV1/FVC (%)	0.8605	0.06350		0.010	0.840-0.881
Evening	FEV1 (L)	2.9543	0.52285		0.083	2.787-3.121
Evening	FEV1/FVC (%)	0.8675	0.05109		0.008	0.851-0.884

As elucidated through Table 2, the mean FVC values were 3.426 L in the morning, 3.414 L in the afternoon, and 3.380 L in the evening, with standard deviations of 0.6829491, 0.668947, and 0.695546, respectively. The 95% confidence intervals for FVC were 3.208-3.644 in the morning, 3.200-3.627 in the afternoon, and 3.158-3.602 in the evening, indicating slight decreases throughout the day. However, overlapping confidence intervals suggested these differences were not statistically significant. For FEF25-75%, the mean values were 3.178 L/s in the morning, 3.411 L/s in the afternoon, and 3.389 L/s in the evening, with standard deviations of 1.05981, 0.94953, and 0.77047, respectively. The 95% confidence intervals for FEF25-75% were 2.839-3.517 in the morning, 3.107-3.714 in the afternoon, and 3.142-3.635 in the evening. These values showed an increase from morning to afternoon, followed by a slight decrease in the evening, but overlapping confidence intervals indicated these differences were not statistically significant.

**Table 2:** Evaluation of FVC and FEF (FEF25-75%) at different time intervals FVC: forced vital capacity; FEF25-75%: forced expiratory flow during 25%-75% of expiration; FEF: forced expiratory flow

Time of day	Measure	Mean	Standard deviation	N	Standard error	95% confidence interval (lower bound to upper bound)
Morning	FVC (L)	3.426	0.6829491	40	0.108	3.208-3.644
Afternoon	FVC (L)	3.414	0.668947		0.106	3.200-3.627
Evening	FVC (L)	3.380	0.695546		0.110	3.158-3.602
Morning	FEF25-75% (L/s)	3.178	1.05981		0.168	2.839-3.517
Afternoon	FEF25-75% (L/s)	3.411	0.94953		0.150	3.107-3.714
Evening	FEF25-75% (L/s)	3.389	0.77047		0.122	3.142-3.635

The mean FEV1/PEF values were 0.444 in the morning, 0.455 in the afternoon, and 0.468 in the evening, with standard deviations of 0.081074, 0.050778, and 0.060234, respectively (Table 3). The 95% confidence intervals for FEV1/PEF were 0.418-0.470 in the morning, 0.439-0.471 in the afternoon, and 0.449- 0.487 in the evening. These results indicated a slight increase throughout the day, but overlapping confidence intervals suggested these differences were not statistically significant. The mean salivary cortisol levels were 1.520 ng/mL in the morning, 1.402 ng/mL in the afternoon, and 1.186 ng/mL in the evening, with standard deviations of 0.306278, 0.339732, and 0.387294, respectively. The 95% confidence intervals for salivary cortisol were 1.421-1.619 in the morning, 1.292-1.512 in the afternoon, and 1.061-1.312 in the evening. These values decreased throughout the day, with nonoverlapping confidence intervals indicating that the differences were statistically significant.

**Table 3:** Evaluation of FEV1/PEF and salivary cortisol at different time intervals FEV1: forced expiratory volume in one second; PEF: peak expiratory flow

Time of day	Measure	Mean	Standard deviation	N	Standard error	95% confidence interval (lower bound to upper bound)
Morning	FEV1/PEF	0.444	0.081074	40	0.013	0.418-0.470
Afternoon	FEV1/PEF	0.455	0.050778		0.008	0.439-0.471
Evening	FEV1/PEF	0.468	0.060234		0.010	0.449-0.487
Morning	Salivary cortisol (ng/mL)	1.520	0.306278		0.049	1.421-1.619
Afternoon	Salivary cortisol (ng/mL)	1.402	0.339732		0.054	1.292-1.512
Evening	Salivary cortisol (ng/mL)	1.186	0.387294		0.062	1.061-1.312

The Pearson correlation between salivary cortisol levels and FVC was .829 (p = 0.055), showing a strong positive correlation approaching significance (Table 4). For FEV in one second (FEV1), the correlation was -0.649 (p = 0.038), indicating a moderate negative and statistically significant correlation. The FEV1/FVC ratio had a correlation of -0.730 (p = 0.040), showing a strong negative and significant correlation. In contrast, the correlation with FEF was weak and negative at -0.142 (p = 0.383), and with PEF was negligible at 0.030 (p = 0.857), indicating no significant correlation. The FEV1/PEF ratio had a weak positive correlation of 0.120 (p = 0.460), also not significant.

**Table 4:** Correlation between salivary cortisol levels and various pulmonary function measures among the study group FVC: forced vital capacity; FEV1: forced expiratory volume in one second; FEF: forced expiratory flow; PEF: peak expiratory flow

Measure	Pearson correlation (salivary cortisol)	Sig. (two-tailed)	N
FVC	0.829	0.055	40
FEV1	-0.649	0.038	
FEV1/FVC	-0.730	0.040	
FEF	-0.142	0.383	
PEF	0.030	0.857	
FEV1/PEF	0.120	0.460	

## Discussion

The observed slight increases in FEV1 and FEV1/FVC ratios throughout the day, alongside the significant decrease in salivary cortisol levels, suggest that circadian rhythms may influence respiratory function and endocrine responses. This information is crucial for clinicians and researchers as it highlights the importance of considering the time of day when conducting spirometric assessments and interpreting the results. The significant correlations between salivary cortisol levels and specific spirometric parameters, such as the strong positive correlation with FVC and the negative correlations with FEV1 and FEV1/FVC ratio, indicate a potential physiological link between cortisol secretion and respiratory function. These findings could inform future studies aimed at exploring the underlying mechanisms driving these correlations and their clinical relevance.

Salivary cortisol measurements have significantly contributed to understanding the hypothalamic-pituitary-adrenal axis and its regulation of the autonomic nervous system. However, this method presents several limitations. These include variability in saliva sample collection, participant noncompliance with the timing of collection, the number of samples collected, and differences in analytical methodologies [14]. Despite these challenges, salivary cortisol concentrations have been shown to correlate well with serum-free cortisol levels, providing reliable estimates of serum-free cortisol [15]. Furthermore, salivary cortisol measurement offers certain advantages over serum cortisol measurement. Salivary cortisol primarily reflects the free, biologically active fraction of cortisol, as it is not bound to corticosteroid-binding globulin. This method is also noninvasive and straightforward, reducing ethical concerns and the burden on participants.

A common issue in the literature is the frequency of saliva sampling. Some studies have indicated that frequent sampling, such as every 10 minutes, is necessary to accurately capture the pulsatile nature of plasma cortisol [16]. In contrast, other studies have suggested that less frequent, such as eight-hourly salivary cortisol measurements, can reliably estimate 24-hour cortisol exposure for population studies [17].

Abd-Elaleem et al. [6] examined spirometric changes in overweight individuals with central obesity compared to healthy controls, observing significantly lower spirometric parameters and further reductions in the supine position. While their study focused on the impact of obesity and posture on respiratory function, our study was centered on diurnal variations in a healthy population. Both studies, however, contribute to the broader understanding of factors influencing spirometric outcomes. Goyal et al. [8] explored circadian variability in airway caliber using spirometry at multiple time points throughout the day. They found significant sinusoidal patterns in spirometric parameters, with minimum values at night and maximum values during the day. Similarly, our study found slight increases in spirometric measures during the day, although we did not assess nighttime values. Both studies illustrate the dynamic nature of pulmonary function over 24 hours.

When comparing our findings to those of Kobayashi et al. [11], both studies examined diurnal variations in salivary cortisol. While Kobayashi et al. focused on the distribution characteristics of cortisol and secretory immunoglobulin A (S-IgA), finding significant changes in skewness and kurtosis of cortisol concentrations from morning to afternoon, our study did not delve into distribution characteristics but rather the correlation with spirometric parameters. Both studies underscored the importance of considering diurnal variations in salivary biomarkers. Cvijetic et al. [12] investigated the relationship among salivary cortisol, body composition, and heart rate variability, finding significant associations between cortisol levels and body fat, muscle mass, and bone mass. Their study highlighted the endocrine and metabolic implications of cortisol, akin to our findings of cortisol's impact on respiratory parameters. However, our study was more focused on respiratory function rather than body composition and cardiovascular indices.

Chronesthesy is an emerging concept in pharmacology that describes rhythm-dependent variations in medication effects, which cannot be solely attributed to pharmacokinetics. These variations arise from rhythmic changes in the free fraction of drugs, receptor numbers and conformations, dynamics of second messengers, and rate-limiting steps in metabolic pathways within drug-targeted tissues. Examples of medications affected by chronesthesy include analgesics, anticoagulants, beta-adrenergic receptor agonists and antagonists, corticosteroids, and nonsteroidal anti-inflammatory drugs [18-20].

Circadian variations in respiratory physiology are well documented in the literature. The suprachiasmatic nucleus, located in the anterior hypothalamus, serves as the primary circadian pacemaker in humans, regulating circadian rhythms of behavior and organ activity [21-23]. Israel was the first to report differences between the diurnal and nocturnal values of FEV1 and FVC in patients with respiratory pathologies (such as asthma or bronchitis) and healthy subjects. In hospitalized patients, nocturnal values of FVC and FEV1 were decreased without worsening symptoms. However, no significant differences were observed in diurnal and nocturnal values in healthy individuals, possibly because the diaphragm's ability to generate pressure might be preserved in COPD patients.

The results of the present study suggest that spirometric parameters exhibit a distinct circadian rhythm. This finding aligns with several other studies [24]. A morning peak was observed in FEV1, FEV1/FVC, FEF25-75%, FEF50, and PEF values. A study by King et al. [25] indicated that lung elasticity significantly influences airway caliber and that reduced airway caliber is associated with aging. Consequently, spirometric parameters assessed in younger populations may differ from those in older populations.

Spirometry is an effective tool for assessing airway caliber. While adults typically experience a gradual decline in lung function as they age, certain occupational and personal exposures can accelerate this loss. Periodic spirometry testing can detect such accelerated declines. Longitudinal evaluation involves measuring baseline lung function and comparing it to follow-up values obtained at later time points to assess lung function loss over time.

The study had several limitations that may have impacted the findings obtained. First, the sample size was relatively small, consisting of only 40 participants, which may have limited the generalizability of the results. Second, the measurements of spirometric parameters and salivary cortisol levels were taken only at three specific time intervals (morning, afternoon, and evening), which may not fully capture the complexity of diurnal variations. The overlap in confidence intervals for many of the spirometric parameters suggested that the observed differences were not statistically significant, possibly due to the limited number of time points assessed. Moreover, environmental factors such as temperature, humidity, and air quality, which can influence respiratory function, were not controlled or recorded during the study. The use of a single testing location might have introduced site-specific biases.

## Conclusions

As per our findings, there were observable, albeit minor, diurnal variations in spirometric parameters among apparently healthy adults, with FEV in one second and FEV1/FVC ratios showing slight increases from morning to evening. The significant decrease in salivary cortisol levels throughout the day indicated a clear circadian influence on cortisol secretion. The correlations identified between salivary cortisol and specific spirometric parameters, such as the positive correlation with FVC and the negative correlations with FEV1 and FEV1/FVC ratio, suggested a potential physiological interplay between respiratory function and cortisol levels. These findings underscore the importance of considering the time of day in respiratory assessments and highlight the need for further research to explore the underlying mechanisms and clinical implications of these diurnal variations.
